# Frameshift Mutations in Exon 1 of *Fgf10* Frequently Yield Morphologically Normal Embryos

**DOI:** 10.3390/jdb14030033

**Published:** 2026-08-01

**Authors:** Khanui Lkhagvadorj, Eiichi Okamura, Toshifumi Morimura, Seiya Mizuno, Masatsugu Ema

**Affiliations:** 1 Department of Stem Cells and Human Disease Models, Research Center for Animal Life Science, Shiga University of Medical Science, Otsu 520-2192, Shiga, Japan; khanui@belle.shiga-med.ac.jp (K.L.); morimura@belle.shiga-med.ac.jp (T.M.); 2 Laboratory Animal Resource Center, Trans-Border Medical Research Center, University of Tsukuba, Tsukuba 305-8575, Ibaraki, Japan; konezumi@md.tsukuba.ac.jp

**Keywords:** CRISPR/Cas9, *Fgf10*, frameshift mutation, genome editing, MMEJ, Netstart 2.0, alternative translation initiation

## Abstract

Background/Objectives: CRISPR/Cas9-mediated genome editing enables efficient generation of knockout mouse models; however, frameshift mutations do not always result in complete loss of function. The factors influencing functional inactivation following frameshift mutations remain incompletely understood. Here, we tested whether frameshift-dominant targeting of exon 1 is sufficient to generate a null allele of *Fgf10*, a gene essential for limb formation. Methods: Guide RNAs (gRNAs) were selected using a machine learning-based pipeline to favor microhomology-mediated end joining (MMEJ)-dominant repair. Editing efficiency and indel profiles were assessed via amplicon sequencing in mouse embryonic stem cells (mESCs) and preimplantation embryos. Edited embryos were transferred to surrogate females and analyzed at embryonic day 15.5 (E15.5). Results: Amplicon sequencing confirmed >97% editing efficiency and >80% frameshift alleles in both mESCs and preimplantation embryos, with a predominant 7 bp deletion. Despite highly efficient frameshift-dominant editing, most of the E15.5 embryos were morphologically normal, indicating that exon 1 targeting did not reliably produce null phenotypes. In silico analysis suggested the possible presence of alternative downstream translation initiation sites, and our secretion assay supported this possibility. Initiation from a downstream ATG in a +2 reading frame (e.g., 7 bp deletion) may restore the downstream coding sequence and partially preserve protein function. Conclusions: Frameshift mutations in exon 1 of *Fgf10* do not consistently result in functional knockout. The functional outcome depends on the specific reading frame and may be influenced by alternative translation initiation and protein domain architecture. These findings highlight important considerations for designing genome editing strategies to achieve complete gene inactivation.

## 1. Introduction

Generating a knockout (KO) mouse model has become technically straightforward, and highly efficient genome editing can now be routinely achieved [1]. This efficiency is largely attributable to the activation of endogenous DNA repair pathways following Cas9-induced double-strand breaks, which enable precise genome modifications. Specifically, homology-directed repair enables targeted knock-in by incorporating a donor template at the site of a double-strand break [2]. Non-homologous end joining is an error-prone repair pathway that often generates heterogeneous and unpredictable indel patterns [3]. While it can produce knockout alleles, the resulting mutations are frequently mosaic and allelically diverse [4]. Alternatively, microhomology-mediated end joining (MMEJ) utilizes short homologous sequences flanking the double-strand break to align DNA ends prior to ligation, frequently resulting in predictable deletions spanning the intervening sequence [5]. In fact, a recently reported CRISPR editing strategy combining in silico prediction of repair outcomes enables the selection of gRNAs that preferentially induce MMEJ, thereby facilitating the generation of mice carrying predictable deletion alleles [6].

Despite these advances in editing precision, achieving a truly functionally null allele remains biologically complex. Gene structures vary considerably in exon organization, alternative start codons, and domain architecture. Consequently, even complete frameshift mutations do not necessarily guarantee complete loss of gene function [7,8,9,10]. The widespread assumption that a frameshift mutation equates to a functional knockout may therefore oversimplify the relationship between genotype and phenotype.

The *Fgf10* gene plays a critical role in limb development and has been widely used as a benchmark locus for functional knockout studies [11,12]. Structurally, FGF10 consists of an N-terminal signal peptide (aa 1–37), which is cleaved during secretion, followed by a flexible N-terminal extension (aa ~38–63) and a conserved β-trefoil core (aa ~64–209) [13,14,15]. The β-trefoil fold, composed of 12 antiparallel β-strands (β1–β12), forms the receptor-binding scaffold required for interaction with FGFR2b and heparan sulfate. While the entire fold contributes to stability, receptor specificity and high-affinity binding are mediated predominantly by central and C-terminal strands, particularly β6–β12. Previous targeting of exon 1, corresponding to the signal peptide and β-trefoil repeats 1–3, resulted in approximately 50–60% limb defect penetrance. It was proposed that incomplete phenotypic penetrance was attributable to in-frame mutations at the target site, which preserved the β-trefoil fold and consequently retained partial FGF10 activity [16]. However, it remains unclear whether generating predominantly homogeneous frameshift mutations in exon 1 is sufficient to fully abolish FGF10 function and achieve complete penetrance.

In the present study, we targeted exon 1 of *Fgf10* using our predictive MMEJ-based pipeline [6] to determine whether frameshift mutations are sufficient to ensure complete functional knockout.

## 2. Materials and Methods

### 2.1. In Silico Prediction of Indel Patterns and gRNA Activity

Indel pattern prediction was performed using the inDelphi web tool (batch mode) [17]. Target exon sequences were submitted as sequence context. For analysis parameters, the Cas9 PAM sequence was set to NGG, and the cell type was specified as mouse embryonic stem cells (mESCs). The output included predicted indel frequency distributions, precision scores, microhomology (MH) strength, and frameshift frequencies for all potential gRNA target sites within the submitted sequence. MH strength is a composite metric summarizing the contribution of local microhomology sequences, determined by their length, GC content, and distance from the cut site. To estimate gRNA editing efficiency, the CRISPRon (v1.0) [18] platform was employed. Editing efficiency denotes the predicted ability of a guide RNA to induce Cas9-mediated cleavage and subsequent mutagenesis at the target site.

### 2.2. Genome Editing in Mouse Embryonic Stem Cells

C57BL/6J mESCs [19] were obtained from the RIKEN BioResource Research Center (Tsukuba, Ibaraki, Japan) and maintained as previously described [20]. Cells were cultured in ESC medium consisting of DMEM (10565-018; Thermo Fisher Scientific, Waltham, MA, USA) supplemented with knockout serum replacement (KSR), 1 mM sodium pyruvate, 0.1 mM 2-mercaptoethanol (M3148; Sigma-Aldrich, St. Louis, MO, USA), 1× non-essential amino acids (11140-050; Thermo Fisher Scientific), 1 mM L-glutamine, 1000 U/mL leukemia inhibitory factor (LIF), 3 mM CHIR99021, and 1 mM PD0325901.

For transfection, 1.2 × 10^6^ mESCs were plated in 24-well plates and transfected with PX458 (pSpCas9(BB)-2A-GFP) plasmids [21] encoding gRNA and Cas9-2A-EGFP using Lipofectamine 2000 (Thermo Fisher Scientific), in accordance with the manufacturer’s instructions. Complementary oligonucleotides containing BbsI-compatible overhangs were designed for gRNA cloning as follows: forward, 5′-caccGCAAGAGCTTGGCAGGTGACA-3′; reverse, 5′-aaacTGTCACCTGCCAAGCTCTTGC-3′. The PX458 plasmid was originally deposited by Feng Zhang (Addgene plasmid #48138) [21].

Forty-eight hours post-transfection, cells were stained with 7-aminoactinomycin D (7-AAD) to assess viability. GFP-positive and 7-AAD-negative cells were isolated by fluorescence-activated cell sorting (FACS) using a FACSAria Fusion flow cytometer (BD Biosciences, San Jose, CA, USA). Genomic DNA was extracted from sorted cells using the NaOH lysis method.

Fluorescence and bright-field images were acquired using a fluorescence microscope, and images were processed using FIJI (ImageJ, v2.16.0) [22]. For figure preparation, minimum and maximum display levels were adjusted uniformly across the entire image. No nonlinear adjustments were applied.

### 2.3. Animal Experiments

C57BL/6JJmsSlc and Slc:ICR mice were purchased from Japan SLC (Hamamatsu, Shizuoka, Japan). Animals were maintained under specific pathogen-free conditions at 23.0 ± 3.0 °C with 50.0 ± 20.0% relative humidity and a 12 h light/dark cycle.

Fertilized oocytes were generated by in vitro fertilization (IVF). Briefly, oocytes were collected from 3-week-old C57BL/6JJmsSlc females following superovulation induced by intraperitoneal administration of CARD HyperOva (Kyudo, Tosu, Japan) and human chorionic gonadotropin. Spermatozoa were harvested from the cauda epididymides of 8–16-week-old C57BL/6JJmsSlc males and preincubated in Fertiup Mouse Sperm Preincubation Medium (Kyudo). Fertilization was performed in HTF medium at 37 °C under 5% CO_2_ for 3–6 h. Presumptive zygotes were washed and cultured in KSOM (Ark Resource, Kumamoto, Japan) until electroporation.

Electroporation was conducted in Opti-MEM I (Thermo Fisher Scientific) containing Cas9 ribonucleoprotein (RNP) complexes composed of crRNA:tracrRNA duplexes and HiFi Cas9 protein (Integrated DNA Technologies, Coralville, IA, USA). Electroporation was performed using a NEPA21 electroporator (Nepa Gene, Ichikawa, Japan) with a 1 mm gap platinum electrode (BEX, Tokyo, Japan). The poring pulse parameters were as follows: 30–40 V, 3.5 ms pulse width, 50 ms interval, four pulses, and 10% attenuation with positive polarity. The transfer pulse parameters were 5 V, 50 ms pulse width, 50 ms interval, five pulses, and 40% attenuation with alternating polarity.

Following electroporation, one-cell embryos were cultured in KSOM to the two-cell stage and transferred into pseudopregnant ICR females or cultured in vitro to the blastocyst stage (E4.5). Embryos at E15.5 were recovered from euthanized surrogate females. Genomic DNA was isolated from the tail and perianal tissues using phenol–chloroform extraction. For blastocyst analysis, 36 embryos were pooled and genomic DNA was extracted via lysis using NaOH.

### 2.4. Target Amplification, Library Preparation, and iSeq 100 Sequencing

For blastocyst and mESC samples, target regions were amplified for amplicon sequencing using a nested PCR approach performed on a T100 thermal cycler (Bio-Rad, Hercules, CA, USA). Primer pairs were designed to generate a 279 bp amplicon spanning the CRISPR cleavage site. The first-round PCR primers consisted of locus-specific sequences targeting *Fgf10* exon 1 (forward: 5′-TGAGACAATTTCCAGTGCCG-3′; reverse: 5′-CTTCTCCAGCGGACATCTCC-3′) fused at their 5′ ends to universal Illumina overhang adapter sequences (forward overhang: 5′-TCGTCGGCAGCGTCAGATGTGTATAAGAGACAG-3′; reverse overhang: 5′-GTCTCGTGGGCTCGGAGATGTGTATAAGAGACAG-3′). During the second round of PCR, dual-index barcodes were incorporated using custom indexing primers synthesized by Eurofins Genomics (Tokyo, Japan) based on Illumina adapter sequences.

PCR products were verified by electrophoresis on 2% agarose gels. Amplicons were purified using a Gel/PCR Purification Mini Kit (Favorgen Biotech Corp., Ping-Tung, Taiwan) followed by AMPure XP bead cleanup (Beckman Coulter Life Sciences, Indianapolis, IN, USA). Purified libraries were subjected to next-generation sequencing using the iSeq 100 platform (Illumina, San Diego, CA, USA), in accordance with the manufacturer’s instructions.

Sequencing data were analyzed with CRISPResso2 (v2.0) [23] to quantify genome editing outcomes, including indel frequency and distribution.

For E15.5 embryo samples, genomic DNA was amplified using primers flanking the same 279 bp target region. PCR products were subjected to Sanger sequencing, and editing efficiency and indel spectra were analyzed using ICE (Inference of CRISPR Edits) software (v3.0, EditCo Bio, Redwood City, CA, USA, 2025).

### 2.5. Statistical Analysis

To assess the relationship between editing outcomes and limb phenotypes, embryos were grouped as limbless, limb defects, or normal based on morphological evaluation. The proportions of in-frame, +1-frame, and +2-frame alleles were compared among phenotypic groups using the Kruskal–Wallis test followed by Dunn’s multiple-comparisons test. Adjusted *p*-values were used for pairwise comparisons, and *p* < 0.05 was considered statistically significant. Statistical analyses were conducted using GraphPad Prism version 11.0.2 (GraphPad Software).

### 2.6. In Silico Prediction of Translation Initiation Sites

Putative translation initiation sites (TISs) were predicted using NetStart 2.0 [24]. The full-length cDNA sequence of the target gene was submitted to the tool using default parameters.

NetStart 2.0 provides a prediction score (0–1) for each candidate start codon, with higher scores indicating greater confidence and reliability. Predicted sites were evaluated based on their respective scores.

### 2.7. FLAG-Tagged Secretion Assay and Western Blot Analysis

#### 2.7.1. Plasmid Construction

Wild-type and mutant mouse Fgf10 coding sequences were amplified from cDNA derived from gRNA-injected fetuses and cloned into pcDNA3.1(+) (Thermo Fisher Scientific, Waltham, MA, USA) to generate C-terminal FLAG-tagged expression constructs.

#### 2.7.2. Cell Culture and Western Blotting

Chinese hamster ovary cells were maintained in Dulbecco’s Modified Eagle Medium (Nacalai Tesque, Kyoto, Japan) supplemented with 10% fetal bovine serum (Sigma-Aldrich, St. Louis, MO, USA) and penicillin/streptomycin (Nacalai Tesque). For transfection, cells were seeded at 40–60% confluency and transfected with 1 μg plasmid DNA per well using Viofectin (Viogene, Taipei, Taiwan). After 24 h, the culture medium was removed, and cells were washed three times with serum-free DMEM, followed by incubation in serum-free DMEM for an additional 24 h. Conditioned medium was then collected, centrifuged to remove cellular debris, and mixed with SDS–PAGE sample buffer. Corresponding whole-cell lysates were prepared in SDS–PAGE sample buffer using equal sample volumes. Protein samples from conditioned medium and cell lysates were analyzed by SDS–PAGE and Western blotting using rabbit anti-FLAG antibody (anti-FLAG antibody (#14793)).

#### 2.7.3. Immunocytochemistry

Transfected cells were fixed with 4% paraformaldehyde for 10 min, permeabilized with 0.1% Triton X-100, and incubated with mouse anti-FLAG (M2; Sigma-Aldrich, St. Louis, MO, USA) and rabbit anti-GRP78 (ab21685; Abcam, Cambridge, UK) antibodies for 1 h at room temperature. Cells were then incubated with CF488-conjugated donkey anti-mouse IgG (Biotium, Fremont, CA, USA) and Alexa Fluor 647-conjugated donkey anti-rabbit IgG (Thermo Fisher Scientific, Waltham, MA, USA). Nuclei were counterstained with DAPI. Images were acquired using a Leica Stellaris 8 (Leica Microsystems, Wetzlar, Germany).

## 3. Results

### 3.1. In Vitro Validation of inDelphi-Guided gRNA Targeting of Fgf10 Exon 1

**Figure 1:**
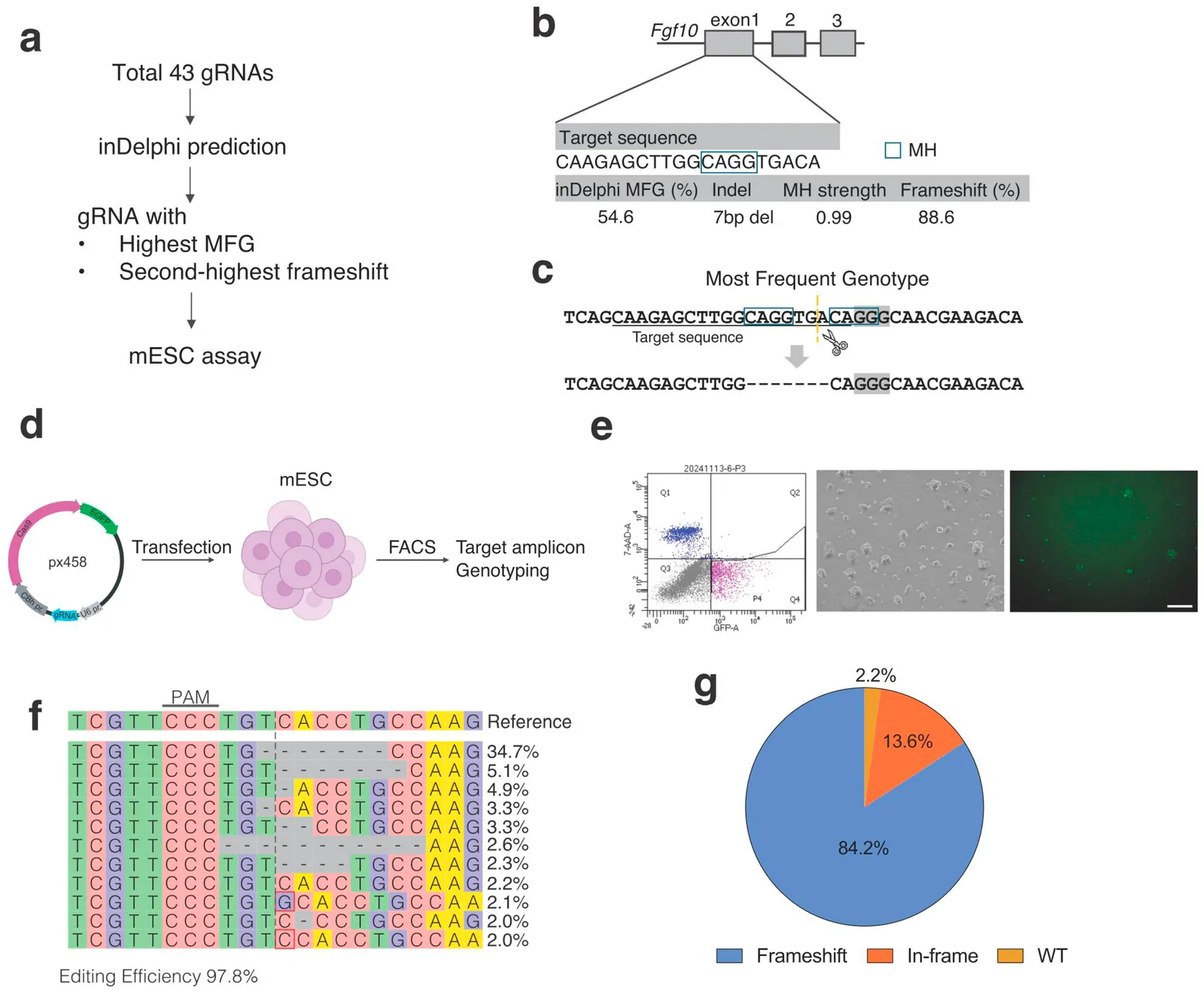
In vitro validation of inDelphi-guided gRNA targeting of *Fgf10* exon 1. (**a**) Workflow of the experimental procedures. (**b**) Schematic representation of the mouse *Fgf10* locus showing gRNA target sites within exon 1. Corresponding inDelphi predictions are indicated. Blue boxes denote microhomology (MH) sequences. (**c**) Schematic illustration of the most frequent predicted genotype utilizing a 4 bp microhomology to generate a 7 bp deletion. (**d**) Workflow of the mESC genome editing experiment (Created in BioRender. Okamura, E. (2026) https://BioRender.com/sf4nb3u). (**e**) Representative bright-field (BP) and GFP images of mESCs. FACS gating strategy is shown; P4 indicates GFP-positive and 7-AAD-negative cells, which were sorted. Fluorescence images were processed in FIJI (ImageJ) for figure preparation by uniformly adjusting the minimum and maximum display levels across the entire image to optimize visualization. (**f**) Representative genotype distribution of edited mESCs. Red boxes indicate insertion. (**g**) Pie graph showing the frequency of frameshift mutations in mESCs. Scale bar = 200 μm.

We identified 43 candidate target sites containing an NGG PAM sequence within exon 1 of the *Fgf10* gene (Figure 1a). The predicted mutational outcomes were analyzed using inDelphi [17], which provides predicted genotype frequency, MH strength, and overall frameshift frequency (Table S1). Among the candidates, we selected a gRNA for which the most frequent genotype (MFG) was predicted to be a 7 bp deletion at a frequency of 54.6%, mediated by a 4 bp MH sequence to promote MMEJ repair (MH strength = 0.99) (Figure 1b,c). The predicted overall frameshift frequency was 88.6%. Notably, this candidate showed the highest predicted precision (54.6%) and the second-highest overall frameshift frequency among the candidates (Table S1). Editing efficiency was further evaluated using CRISPRon (v1.0) [18], which predicted 47% activity. Off-target potential was assessed using CRISPOR (v5.2) [25], yielding an MIT specificity score of 58 and a CFD score of 69, indicating low predicted off-target activity.

To experimentally validate these predictions, mESCs were transfected with PX458 plasmids expressing the selected gRNA (Figure 1d). GFP-positive cells were collected by fluorescence-activated cell sorting (FACS) for downstream analysis. A sufficient number of sorted cells were obtained for genotyping analysis (Figure 1e).

The predicted 7 bp deletion was the predominant allele, accounting for 35% of total mutations (Figure 1f). A second distinct 7 bp deletion was observed at a frequency of 5.1%, followed by a 1 bp deletion at 4.9%. Notably, 84.1% of all mutations resulted in frameshifts (Figure 1g), demonstrating a strong bias toward disruptive alleles consistent with inDelphi predictions. Genotyping revealed exceptionally high overall editing efficiency, with only 2.2% wild-type (WT) sequences detected. This efficiency substantially exceeded the CRISPRon-predicted activity, indicating highly effective genome editing. Together, these results demonstrate that the selected gRNA enables efficient editing and predictably frameshift-dominant editing in mESCs.

### 3.2. Efficient and Predictable Genome Editing in Preimplantation Embryos

**Figure 2:**
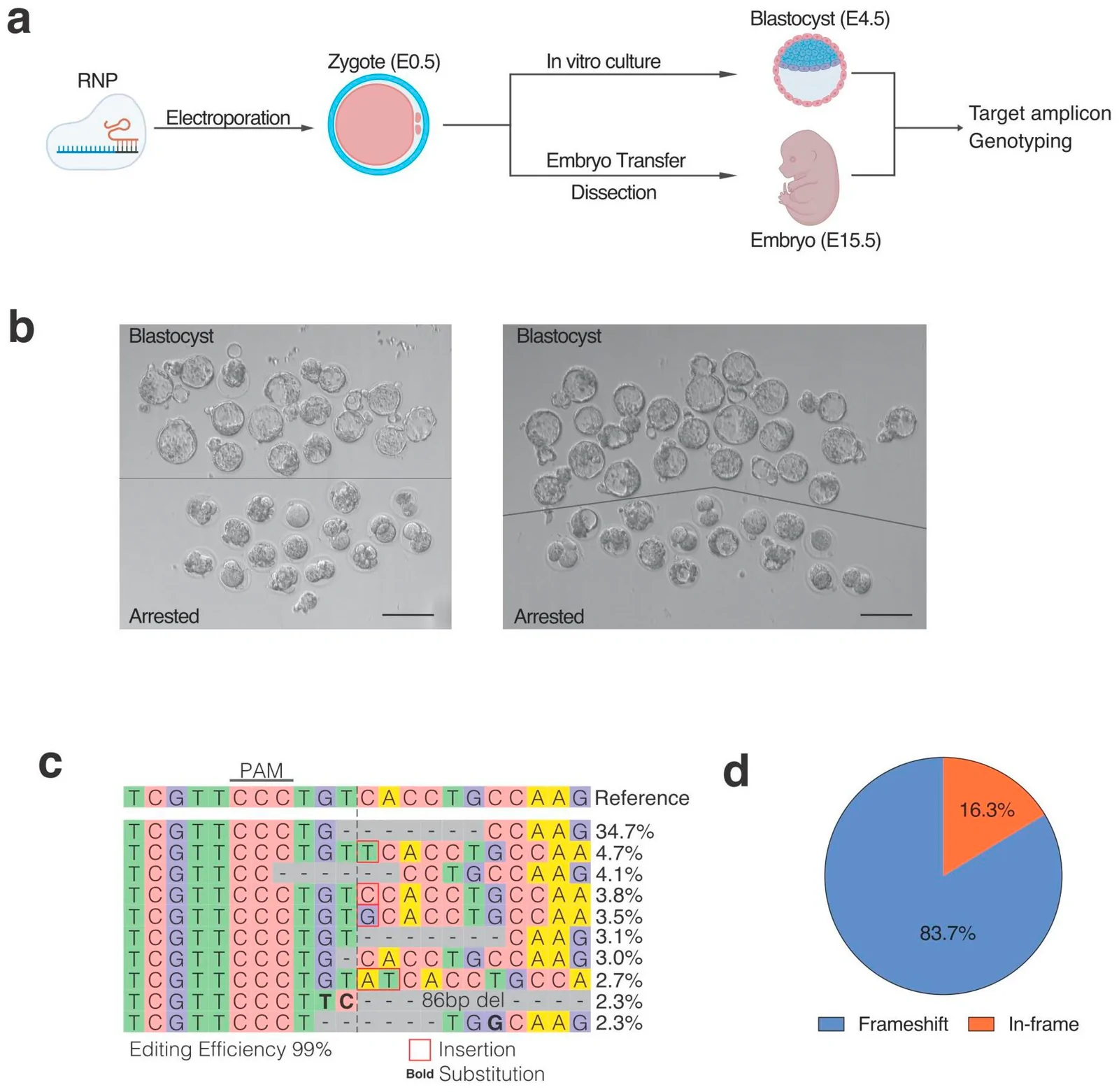
Predictable genome editing in preimplantation embryos. (**a**) Workflow of the experimental procedures (Created in BioRender. Okamura, E. (2026) https://BioRender.com/sf4nb3u). (**b**) Electroporated embryos at the blastocyst stage from two independent experiments. Upper panels show normally developed blastocysts selected for genotyping, and lower panels show arrested embryos. Genomic DNA from embryos of both experiments was pooled and subjected to bulk DNA sequencing in a single reaction. (**c**) Representative genotype distribution of edited blastocysts. Red boxes indicate insertion, and bold text indicates substitution. (**d**) Pie graph showing the frequency of frameshift mutations in mESCs. Scale bar = 200 μm.

To evaluate editing outcomes in embryos, in vitro fertilization was performed using C57BL/6JmsSlc mice (Figure 2a). A total of 67 zygotes were electroporated with the gRNA–Cas9 ribonucleoprotein (RNP) complex, and 36 embryos (54%) developed to the blastocyst stage (Figure 2b). Bulk DNA sequencing of all blastocysts revealed approximately 100% editing efficiency. Consistent with inDelphi predictions and the mESC results, the 7 bp deletion was the predominant genotype (34.7%), followed by a 1 bp insertion (4.7%) (Figure 2c). Frameshift mutations accounted for 83.7% of all alleles (Figure 2d). The overall allele distribution showed a clear predominance of the predicted 7 bp deletion, indicating highly efficient and reproducible editing at the population level. Together, these findings demonstrate that the selected gRNA enables exceptionally efficient, predictable, and frameshift-dominant genome editing in preimplantation embryos.

### 3.3. Morphological Assessment of F_0_ Embryos Reveals Discordance Between Genotype and Morphology

To assess morphologic outcomes, edited embryos were transferred into two recipient females and dissected at E15.5. As *Fgf10* knockout is known to cause multiple developmental abnormalities, including lung agenesis and severe limb defects [11,12,26,27], we focused on limb morphology as the primary readout, as these features are externally visible and robust indicators of *Fgf10* loss of function.

**Figure 3:**
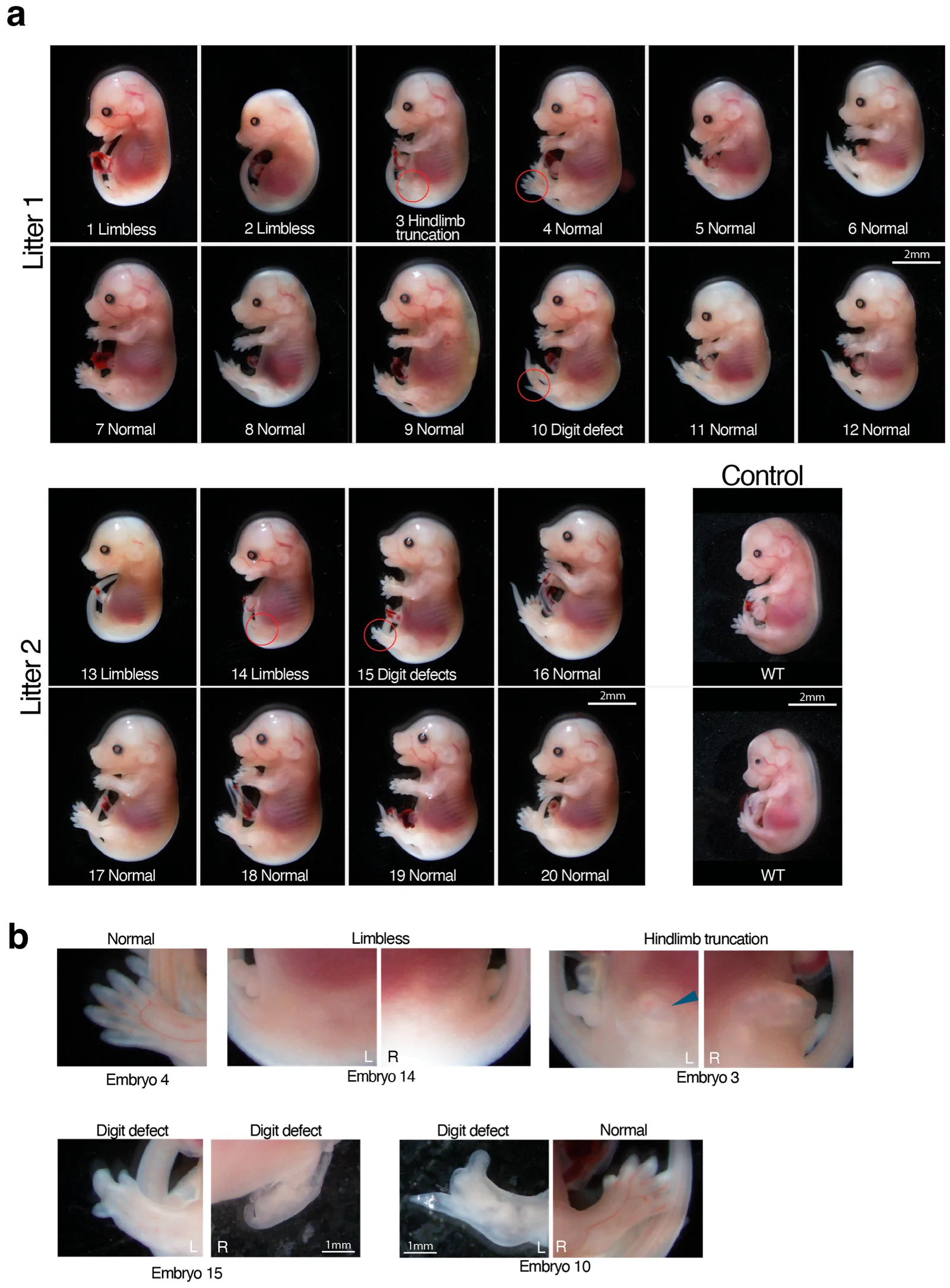
Morphological assessment of CRISPR-edited E15.5 embryos. (**a**) Whole-mount images of edited E15.5 embryos. Embryos 1, 2, 13, and 14 are limbless. Embryo 3 shows hindlimb truncation, while Embryos 10 and 15 exhibit hindlimb digit defects. The remaining embryos (*n* = 13) display morphologically normal limbs comparable to WT embryos (*n* = 2). Red circles indicate the regions shown at higher magnification in panel (**b**). Scale bar = 2 mm. (**b**) Higher-magnification views of representative normal, limbless, hindlimb truncation, and hindlimb defect phenotypes shown in (**a**). Blue arrows indicate limb buds. Scale bar = 2 mm.

In litter 1 (*n* = 12), eight embryos exhibited normal limb morphology, two (Embryos 1 and 2) were completely limbless, Embryo 3 showed hindlimb truncation, and Embryo 10 showed a left hindlimb digit defect (Figure 3 and Figure S1). In litter 2 (*n* = 8), five embryos exhibited normal limb morphology, two (Embryos 13 and 14) were limbless, and one (Embryo 15) displayed digit defects in both hindlimbs compared to WT controls.

Analysis of allele composition in individual embryos revealed limited allelic complexity overall. Four embryos (Embryos 11, 13, 18, and 19) exhibited a single detectable allele, while most embryos carried between two and six distinct alleles. One embryo (Embryo 15) displayed higher allelic diversity, with 12 detectable alleles. Notably, 16 embryos harbored the predicted 7 bp deletion among their alleles, supporting the high reproducibility and consistency of the editing outcome across embryos.

**Figure 4:**
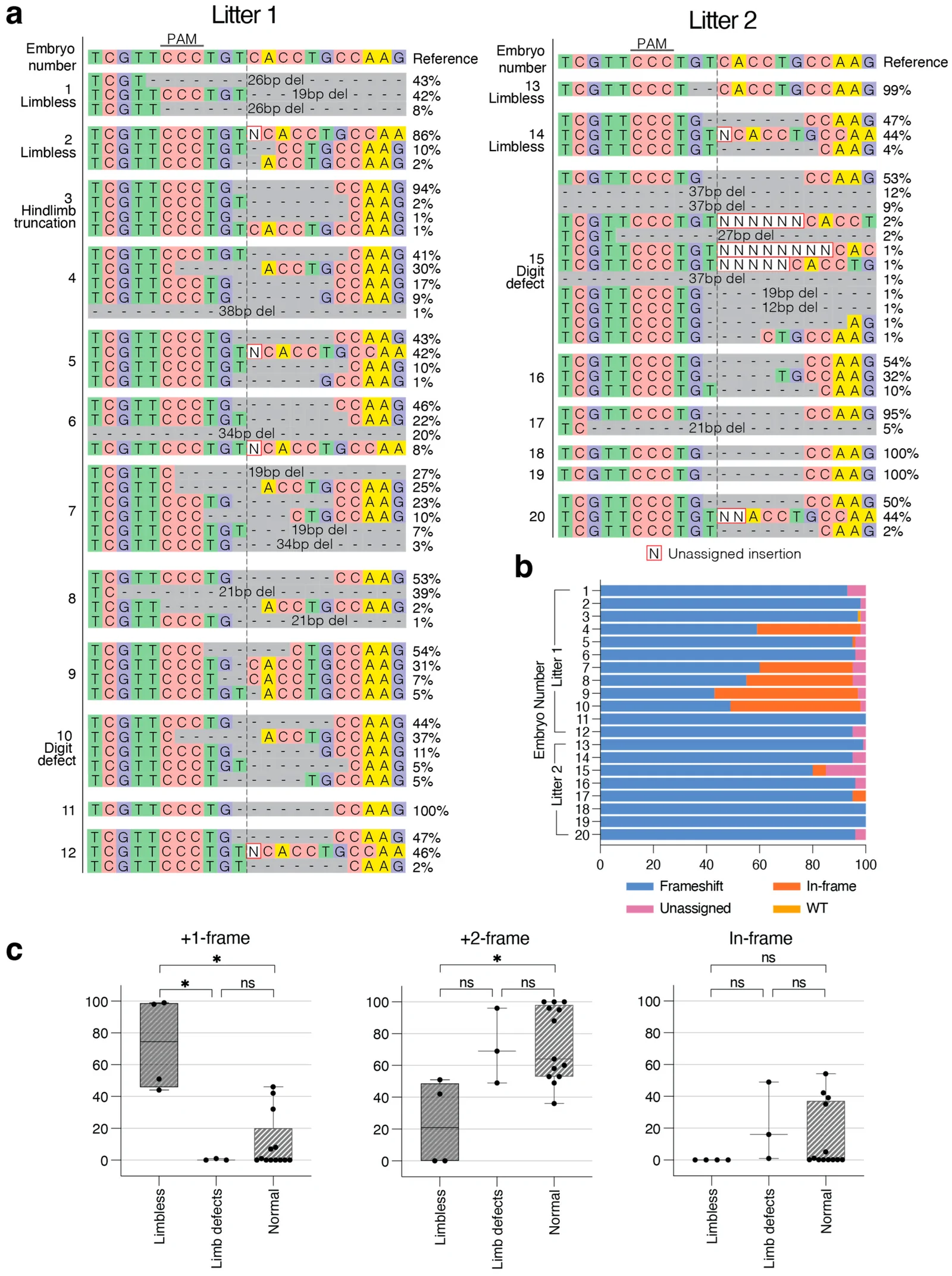
Genotyping analysis of post-implantation embryos. (**a**) Genotype distribution of individual E15.5 embryos. (**b**) Bar graph showing the frequency of frameshift mutations in each embryo as determined by ICE analysis. N (unassigned) represents sequencing signals that could not be confidently decomposed into defined indel alleles due to overlapping peaks or allelic complexity in Sanger sequencing traces. (**c**) Reading-frame composition according to embryo morphology. Embryos were classified as limbless, limb defect, or normal, and the proportions of +1-frame, +2-frame, and in-frame alleles were determined from amplicon sequencing data. Each point represents an individual embryo. Box plots indicate the median and interquartile range, with whiskers showing the full data range. Statistical significance was assessed using the Kruskal–Wallis test followed by Dunn’s multiple-comparisons test. ns, not significant; * *p* < 0.05.

Genotyping analysis revealed an editing efficiency nearing 100% in all embryos. Frameshift mutations constituted more than 90% of the alleles in 15 embryos (Figure 4a,b), whereas 5 embryos showed a higher proportion (approximately 40–60%) of in-frame mutations (Embryos 4 and 7–10); notably, these 5 embryos exhibited a grossly normal limb morphology.

Taking the findings together, despite highly efficient, frameshift-dominant, and reproducible genome editing, the majority of the embryos exhibited morphologically normal limbs, revealing a clear discordance between genotype and expected loss-of-function outcomes.

To further investigate the relationship between genotype and morphology, embryos were classified as limbless, limb defect (hindlimb truncation and digit abnormalities), or normal and stratified according to the proportion of in-frame alleles and frameshift alleles that produced +1- or +2-frame shifts relative to the wild-type coding sequence (Figure 4c). The +1-frame category comprised alleles such as 1 bp insertions and 2 or 5 bp deletions, whereas the +2-frame category was dominated by the recurrent 7 bp deletion.

A clear association emerged between reading-frame composition and limb morphology. Limbless embryos (Embryos 1, 2, 13, and 14) were enriched for +1-frame alleles, whereas morphologically normal embryos were enriched for +2-frame alleles. Significant differences among morphological groups were observed for both +1-frame (*p* = 0.0018) and +2-frame alleles (*p* = 0.0173), as well as for in-frame alleles (*p* = 0.0495), although the latter effect was comparatively weak. Limbless embryos contained significantly higher proportions of +1-frame alleles than either embryos with limb defects (*p* = 0.0241) or morphologically normal embryos (*p* = 0.0119). Conversely, morphologically normal embryos contained significantly higher proportions of +2-frame alleles than limbless embryos (*p* = 0.0253). Embryos with limb defects were not significantly different from morphologically normal embryos for either +1-frame or +2-frame allele frequencies. Together, these findings suggest that enrichment of +1-frame alleles is associated with complete limb loss, whereas enrichment of +2-frame alleles is associated with normal limb development. However, overall reading-frame composition alone does not fully explain the limb-defect phenotype.

### 3.4. In Silico Analysis of Translation Initiation Sites

To investigate the mechanism underlying this phenomenon, particularly the possibility of alternative translation initiation, cDNA sequences were analyzed using NetStart 2.0 [24], which evaluates candidate start codons based on sequence context features and enables comparison of multiple potential initiation sites. NetStart provides prediction scores ranging from 0 to 1, where higher scores indicate a greater likelihood that a given ATG functions as a true initiation site.

**Figure 5:**
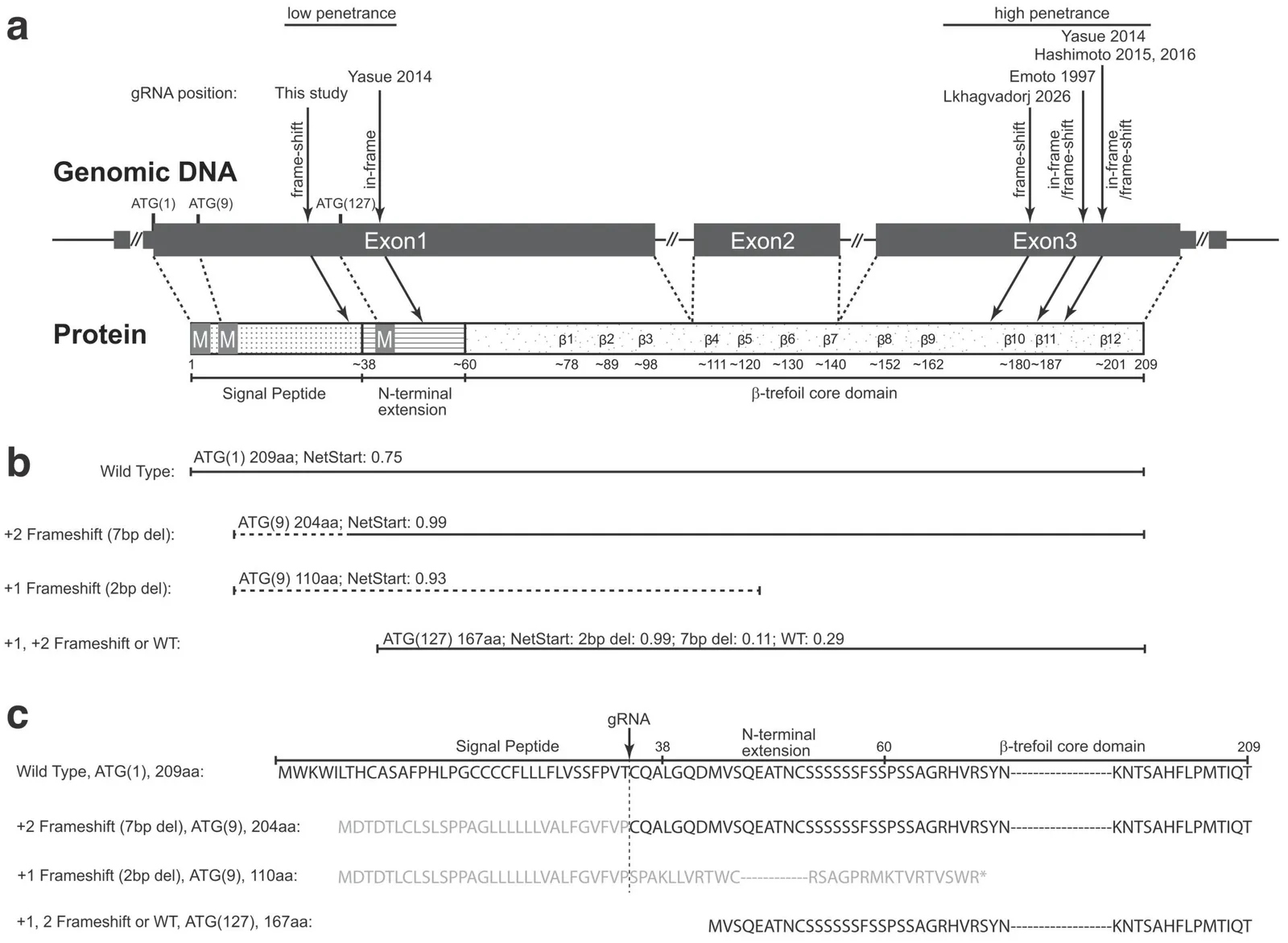
Schematic overview of the Fgf10 gene, protein structure, and translation initiation predictions. (**a**) Schematic representation of the Fgf10 genomic organization and corresponding protein domains. Exon 1 contains the canonical translation start site ATG(1) and encodes the N-terminal signal peptide required for secretion. An alternative in-frame start codon, ATG(127), located downstream of the CRISPR target site, is indicated. Another downstream start codon, ATG(9), is also shown and exhibits high predicted initiation potential in the mutant sequence. The conserved β-trefoil core domain, composed of 12 antiparallel β-strands (β1–β12), is illustrated. This β-trefoil architecture forms a compact and structurally stable scaffold characteristic of FGF family proteins. Target positions reported in this study and previous literature (Yasue et al., 2014 [16]; Lkhagvadorj et al., 2026 [6]; Emoto et al., 1997 [15]; Hashimoto & Takemoto, 2015 [28]; Hashimoto et al., 2016 [29]) are indicated with arrows across exon 1 (low phenotypic penetrance) and exon 3 (high phenotypic penetrance). (**b**) Comparison of NetStart 2.0 prediction scores (range: 0–1, with higher values indicating greater confidence) for WT and 7 bp deletion sequences at ATG(1), ATG(9), and ATG(127), together with the corresponding predicted protein products. (**c**) Amino acid sequence comparison of predicted proteins derived from WT and 7 bp deletion sequences initiated at ATG(1), ATG(9), and ATG(127).

In the WT sequence, initiation from the first start codon (ATG(1); numbers indicate cDNA nucleotide position) was predicted to produce the full-length 209 aa protein, with a score of 0.75. The second start codon (ATG(9)), located eight nucleotides downstream from ATG(1), was predicted to produce a truncated 90 aa protein, with a lower score (0.17). The third start codon (ATG(127)), which is in the same reading frame as ATG(1), was predicted to generate a 167 aa protein (score: 0.29) (Figure 5b,c).

Following the introduction of the most frequent 7 bp deletion, initiation from ATG(1) resulted in a premature stop codon, producing a truncated 111 aa protein (score: 0.28). In contrast, initiation from ATG(9) was predicted to produce a 204 aa protein, in which the N-terminal signal peptide region up to the mutated site differs from the WT, while the downstream amino acid sequence is restored in-frame and becomes identical to the WT, with a higher score (0.99) (Figure 5b,c). Initiation from ATG(127) was predicted to yield a 167 aa protein, identical to the WT-derived product from this site, as it is located downstream of the target site (score: 0.11).

In contrast, +1-frame mutations were observed in limbless embryos, including Embryo 1 (26 bp deletion), Embryo 2 (1 bp insertion and 2 bp deletion), and Embryo 13 (2 bp deletion). As a representative example, we analyzed the 2 bp deletion scenario. Initiation from ATG(1) was predicted to produce a truncated 92 aa protein (score: 0.004), from ATG(9) a 110 aa protein (score: 0.93), and from ATG(127) a 167 aa protein (score: 0.99), identical, as it is located downstream of the target site (Figure 5b,c). Comparison of the amino acid sequences derived from ATG(9) in the 7 bp deletion and 2 bp deletion cases showed identical sequences up to the target site; however, in the 2 bp deletion, a premature stop codon occurs at 110 aa (Figure 5c).

These findings indicate that ATG(9), although upstream of the CRISPR target site, lies in the +2 reading frame relative to ATG(1). Therefore, indels producing a +2 frameshift relative to ATG(1), such as the 7 bp deletion, may preserve the downstream coding sequence in the ATG(9) frame. As a result, although the signal peptide region is altered, the remaining protein sequence can remain largely identical to WT (Figure 5c).

### 3.5. Functional Validation of Alternative Translation Initiation by FLAG-Tagged Localization and Secretion Assays

**Figure 6:**
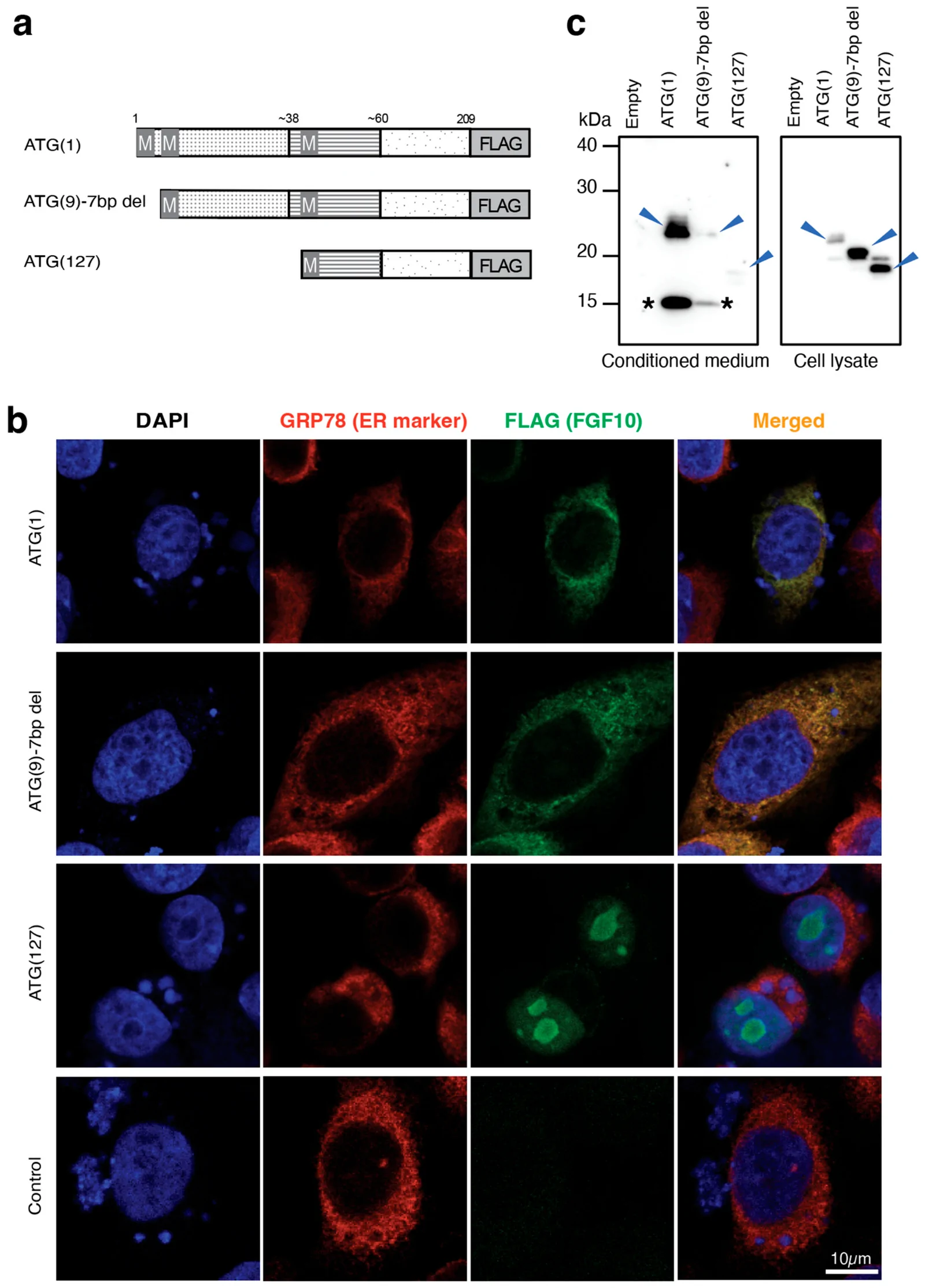
Functional validation of alternative translation initiation by FLAG-tagged localization and secretion assays. (**a**) Schematic of FLAG-tagged ATG(1), ATG(9) 7 bp deletion, and ATG(127) constructs used for functional analysis, showing predicted translation initiation sites and C-terminal FLAG tag insertion. (**b**) Confocal immunofluorescence analysis of FLAG-tagged ATG(1), ATG(9) 7 bp deletion, and ATG(127) constructs transiently expressed in CHO cells. Cells were immunostained with anti-FLAG (green), and the endoplasmic reticulum marker GRP78 (red) and nuclei were counterstained with DAPI (blue). ATG(1) and ATG(9) 7 bp deletion showed strong colocalization with GRP78, indicating preserved ER localization, whereas ATG(127) showed predominantly nuclear localization. Scale bar = 10 μm. (**c**) Blue arrows indicate the major FLAG-positive bands detected in the conditioned medium of ATG(1) WT and ATG(9) 7 bp deletion. Arrowheads and asterisks indicate FLAG-tagged FGF10 proteins and the cleaved products, respectively.

To experimentally evaluate whether translation initiation from the downstream ATG(9) codon could generate a functional signal peptide following the 7 bp deletion, expression constructs corresponding to ATG(1), ATG(9) 7 bp deletion, and ATG(127) were generated with a C-terminal FLAG tag and transiently transfected into CHO cells (Figure 6a).

To examine intracellular localization, transfected cells were immunostained with anti-FLAG and an endoplasmic reticulum (ER) marker GRP78, counterstained with DAPI, and analyzed by confocal microscopy (Figure 6b and Figure S2). FLAG signals from both the ATG(1) and ATG(9) 7 bp deletion showed strong colocalization with GRP78, indicating proper localization to the ER. In contrast, ATG(127) showed predominantly nuclear localization with minimal overlap with GRP78, indicating loss of normal secretory pathway targeting.

Next, conditioned medium was collected following incubation in serum-free medium and analyzed by Western blot using an anti-FLAG antibody (Figure 6c). ATG(1) produced a FLAG-positive band at approximately 25 kDa in the conditioned medium (Figure 6c, arrowhead). Notably, the 25 kDa band detected in the conditioned medium migrated more slowly than the corresponding band detected in the cell lysate, suggesting glycosylation occurred during secretion of the ATG(1) protein. Similarly, ATG(9) 7 bp deletion produced a FLAG-positive band at approximately 25 kDa in the conditioned medium, which also exhibited slower migration compared with the corresponding intracellular protein (Figure 6c and Figure S3), suggesting successful secretion and glycosylation. However, the protein expression level of ATG(9) 7 bp deletion was markedly reduced compared with that of ATG(1), suggesting reduced secretion efficiency. In contrast, the ATG(127) product was detected only at low levels in the conditioned medium, and its molecular weight was similar to that detected in the cell lysate, suggesting that glycosylation did not occur (Figure 6c, arrowhead). These results indicate that proteins derived from ATG(1) and ATG(9) 7 bp deletion are processed through the ER–Golgi secretory pathway, whereas the ATG(127) product may be released through a non-classical secretion pathway or from dead cells.

Together, these results demonstrate that alternative translation initiation from ATG(9) preserves signal peptide function and protein secretion in +2-frame mutants, supporting the hypothesis that downstream translation initiation contributes to the maintenance of normal limb morphology despite CRISPR-induced frameshift mutations.

## 4. Discussion

Frameshift mutations are presumed to produce complete loss-of-function alleles, which may oversimplify the relationship between genotype and phenotype, particularly in genes with modular or structurally resilient domain architecture. The functional complexity of gene disruption is clearly demonstrated in *Fgf10*. FGF10 is an essential mesenchymal growth factor required for limb bud initiation and epithelial–mesenchymal signaling, and the complete loss of FGF10 results in limb agenesis [11].

In a previous study [6,16,28,29], targeting of exon 3 of *Fgf10*, which disrupts the region encoding β8–β12, resulted in a complete null phenotype, consistent with direct impairment of the receptor-binding interface and structural core (Figure 5a). Notably, embryos targeted at *Fgf10* exon 3 frequently exhibited limb-defect morphologies, even when multiple mutant alleles were present, including in-frame indels [29]. These observations indicate that in-frame mutations or allelic mosaicism do not necessarily prevent limb defects when essential functional domains are disrupted.

In contrast, exon 1 targeting produced embryos with apparently normal limb development despite a frameshift mutation in this study. Therefore, the inconsistent occurrence of limb defects in exon 1-targeted embryos is unlikely to be explained solely by residual in-frame alleles or undetected mosaicism.

A more plausible explanation suggested by in silico analysis is that translation initiation site prediction using NetStart 2.0 [24] identified a downstream ATG(9) with high initiation potential in the mutant sequence, which aligns with a +2 reading frame relative to the original start site. Notably, initiation from this site is predicted to generate a protein in which the N-terminal region proximal to the mutation is altered, whereas the downstream coding sequence is restored in-frame and remains identical to the WT protein, including the β-trefoil core. This suggests that alternative initiation at ATG(9) may partially preserve FGF10 protein function despite the frameshift mutation (Figure 5b,c). Consistent with this prediction, our functional analyses demonstrated that the ATG(9) 7 bp deletion construct showed ER localization, and secretion activity, although at reduced levels compared with ATG(1). These findings further indicate that frameshift mutations alone do not necessarily guarantee complete loss of function, and that the exact reading frame generated by each indel should be carefully evaluated, as specific frameshift configurations may retain substantial functional protein activity.

A limitation of this study is that, although our in vitro secretion assays support alternative translation initiation from ATG(9) and preservation of signal peptide function, we did not directly detect the endogenous alternative protein product in mutant embryos or tissues. Therefore, the in vivo abundance, stability, and functional contribution of this alternative protein to limb development remain to be fully determined. Future studies using approaches such as targeted proteomic analyses, detection of endogenous mutant proteins, or genome editing strategies that specifically disrupt ATG(9) will be required to establish the physiological contribution of alternative initiation in vivo.

## 5. Conclusions

In this study, we used a predictive MMEJ-based CRISPR/Cas9 strategy to target exon 1 of *Fgf10*, achieving efficient frameshift-dominant editing. However, many embryos retained normal limb morphology, indicating that frameshift mutations alone do not necessarily generate functional null alleles.

Our findings suggest that this phenomenon may be explained by alternative downstream translation initiation, where specific frameshift configurations (e.g., +2) preserve the downstream coding sequence and substantial protein function. These results highlight the importance of considering alternative translation initiation sites and targeting essential functional domains when designing CRISPR-mediated knockout strategies.

## Data Availability

The data supporting the findings of this study are available within the article and its Supplementary Materials.

## Supplementary Materials

The following supporting information can be downloaded at: https://www.mdpi.com/article/10.3390/jdb14030033/s1, Table S1: inDelphi analysis of gRNAs targeting *Fgf10* exon 1; Figure S1: Morphological assessment of CRISPR-edited E15.5 embryos (same specimens as Figure 3, showing the contralateral side); Figure S2: Low-magnification immunofluorescence images; Figure S3: Western blot analysis of FLAG-tagged ATG(9) and ATG(127) constructs.
