# Supplementary material for: Frameshift Mutations in Exon 1 of Fgf10 Frequently Yield Morphologically Normal Embryos
Source: J Dev Biol. 2026 Aug 1;14(3):33. doi: 10.3390/jdb14030033 (PMC13511607; doi:10.3390/jdb14030033)
Supplement: Supplementary file 1 [file jdb-14-00033-s001.zip › Supplementary Figure.pdf]

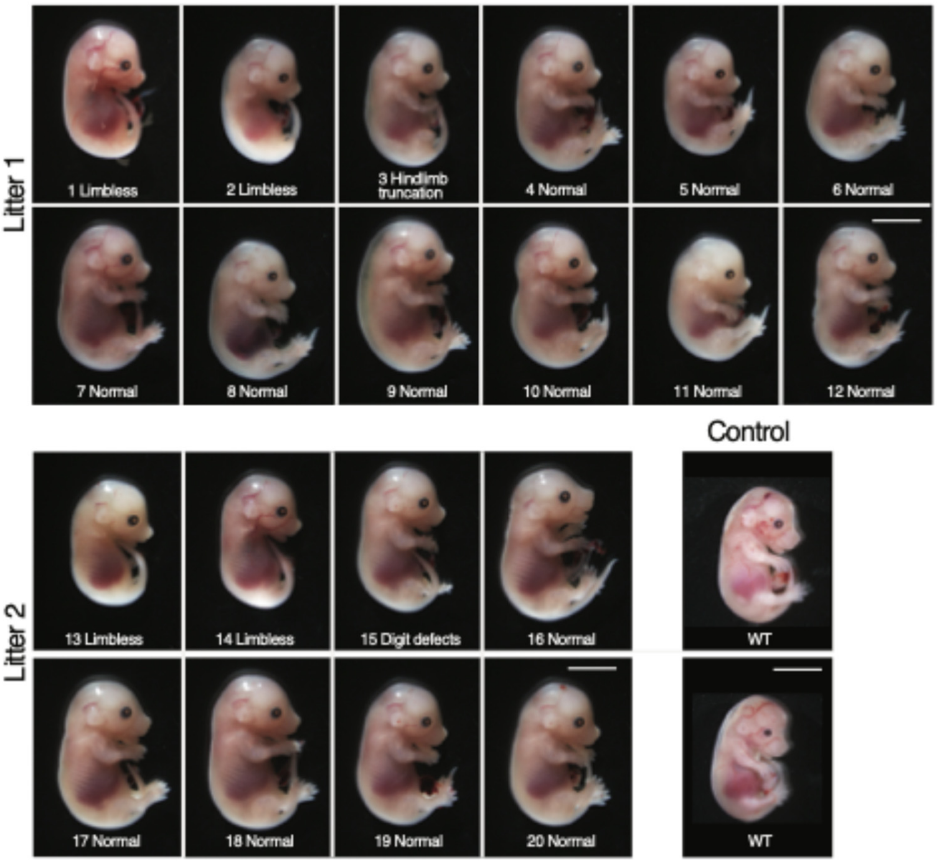

Supplementary Figure S1. Morphological assessment of CRISPR-edited E15.5 embryos (same specimens as Figure 3, showing the contralateral side). Embryos 1, 2, 13, and 14 are limbless. Embryo 3 shows hindlimb truncation, while embryos 10, and 15 exhibit digit defects. Notably, Embryo 10 shows a unilateral digit defect, with a malformed left hindlimb and a morphologically normal right hindlimb. The remaining embryos (n = 13) display normal limb morphology comparable to WT embryos (n = 2). Scale bar = 2 mm.

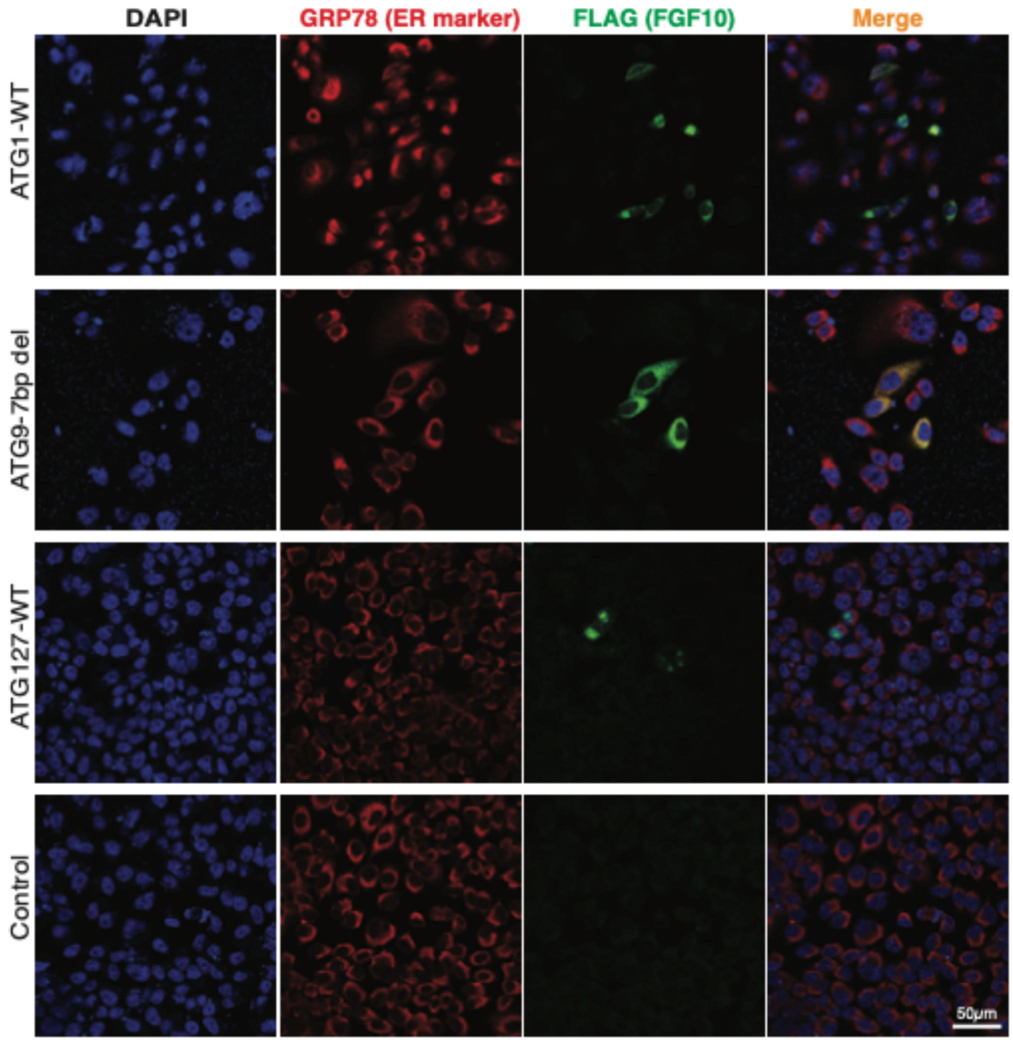

Supplementary Figure S2. Low magnification immunofluorescence images.
Confocal immunofluorescence analysis of FLAG-tagged ATG(1), ATG(9) 7 bp deletion, and ATG(127) constructs transiently expressed in CHO cells. Cells were immunostained with anti-FLAG (green) and the endoplasmic reticulum marker GRP78 (red), with nuclei counterstained with DAPI (blue). Representative wider-field images showing multiple cells confirm the localization patterns observed in Figure 6b. Scale bar = 50 μm.

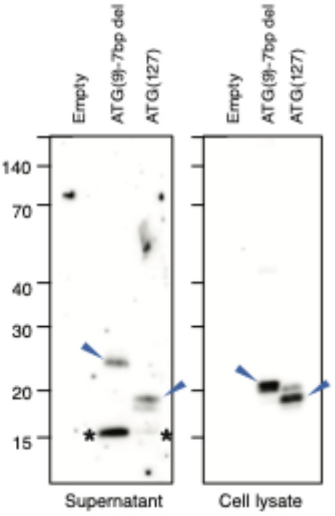

Supplementary Figure S3. Western blot analysis of FLAG-tagged ATG(9) and ATG(127) constructs. Western blot analysis of conditioned medium and corresponding cell lysates collected after transient transfection of FLAG-tagged ATG(9) 7 bp deletion and ATG(127) WT constructs. This experiment represents an independent replicate of the secretion assay shown in Figure 6c. Arrowheads and asterisks indicate FLAG-tagged FGF10 proteins and the cleaved products, respectively.
