# Supplementary material for: Frameshift Mutations in Exon 1 of Fgf10 Frequently Yield Morphologically Normal Embryos
Source: J Dev Biol. 2026 Aug 1;14(3):33. doi: 10.3390/jdb14030033 (PMC13511607; doi:10.3390/jdb14030033)
Supplement: Supplementary file 1 [file jdb-14-00033-s001.zip › Supplementary Table.pdf]

Supplementary Table S1. inDelphi analysis of gRNAs targeting *Fgf10* exon 1. Predicted mutational outcomes include precision score, frameshift frequency, microhomology (MH) strength, and most frequent genotype (MFG) frequency. Predictions were generated using the inDelphi algorithm. MH, microhomology; MFG, most frequent genotype.

gRNA	PAM	Precision	Frameshift (%)	MH strength	MFG (%)
AGCAGCAACAGCAGCCCGGC	AGG	0.56	92.3	1	37.6
CAAGAGCTTGGCAGGTGACA	GGG	0.62	88.6	0.99	54.6
CCAAGAGCTTGGCAGGTGAC	AGG	0.6	88.1	1.04	46.1
GTCCTGACCAAGAGCTTGGC	AGG	0.47	87.6	0.37	19.3
ACCTCCAAGGAGATGTCCGC	TGG	0.5	86.6	0.62	29.3
AGGAGAACAGCCTTCTCCAG	CGG	0.52	84.3	0.75	32.3
TCAGCCTTTCCCCACCTGCC	GGG	0.47	83.8	0.71	17.6
CCAGTGCGGGAAGGCATGTG	CGG	0.59	82.8	1.13	42.7
CCATGTCCTGACCAAGAGCT	TGG	0.43	82.2	0.15	17.2
AGCAACAGCAGCCCGGCAGG	TGG	0.44	81.9	0.71	19.5
CCTCTCCTTCCAGTGCGGGA	AGG	0.52	81.7	0.72	30.7
GCAACAGCAGCCCGGCAGGT	GGG	0.45	81.4	0.67	19.4
CTCAGCCTTTCCCCACCTGC	CGG	0.43	80.2	0.67	20.7
CCTGTCACCTGCCAAGCTCT	TGG	0.4	79.5	0.15	9.5
CAACAGCAGCCCGGCAGGTG	GGG	0.38	77.9	0.63	11.9
GGAGGAAGAAGAGCAGTTGG	TGG	0.47	75.2	0.36	20
ACATGCCTTCCCGCACTGGA	AGG	0.49	74.6	-0.36	17.8
CTTCTCCTCTCCTTCCAGTG	CGG	0.47	74.3	-0.27	14.9
GCAGCCCGGCAGGTGGGGAA	AGG	0.37	74.1	0.81	10.7
AAGGAGATGTCCGCTGGAGA	AGG	0.52	73	0.74	32.6
TCCAGCGGACATCTCCTTGG	AGG	0.47	70.5	0.08	19.5
TCAGGACATGGTGTCACAGG	AGG	0.44	70	0.44	13.5
TTCTCCTCTCCTTCCAGTGC	GGG	0.42	69.9	0.09	10.4
CCGCACATGCCTTCCCGCAC	TGG	0.42	69.3	0.26	8.9
CCAAGCTCTTGGTCAGGACA	TGG	0.49	68.2	0.77	16.8
CTGCTTCTTGTTGCTCTTTT	TGG	0.4	66.9	0.85	10.5
CACCTGCCAAGCTCTTGGTC	AGG	0.44	66.8	0.32	16.6
AGAAGAACGGCAAGGTCAGC	GGG	0.35	66.3	0.72	10
CTCAATCGTGAGAAAGTACT	TGG	0.43	65.3	0.48	13.1
AAGAAGCAGCAACAGCAGCC	CGG	0.5	65.1	0.85	21.3
CGCACTGGAAGGAGAGGAGA	AGG	0.52	63.4	1.74	25.8
GAGAAGAACGGCAAGGTCAG	CGG	0.37	60.7	0.75	10.5
CCTTCCCGCACTGGAAGGAG	AGG	0.49	60.3	1.09	12.8
TGGTCAGGACATGGTGTCAC	AGG	0.49	59.4	0.44	18.9
CGAGGAGGAAGAAGAGCAGT	TGG	0.48	58	0.54	21.8
TTCTCCAGCGGACATCTCCT	TGG	0.48	57.1	0.49	21.1
CGGAGCTACAATCACCTCCA	AGG	0.51	55	0.64	25.8
CACGATTGAGAAGAACGGCA	AGG	0.36	54.7	0.48	13.9
CGGCAGGTGGGGAAAGGCTG	AGG	0.41	54	0.71	32.4
CGTGAGAAAGTACTTGGTGA	AGG	0.46	49.2	0.67	19.6
TTTCTCACGATTGAGAAGAA	CGG	0.53	47.7	0.91	40.2
GGAAGGAGAGGAGAAGGACG	AGG	0.51	32.5	1.71	29.9
AGGAGAGGAGAAGGACGAGG	AGG	0.5	31.6	1.63	24.9
